# Effect of Pregnancy on Anti-HEV Antibody Titres, Plasma Cytokines and the Corresponding Gene Expression Levels in the PBMCs of Patients Presenting with Self-Recovering Clinical and Subclinical Hepatitis E

**DOI:** 10.1371/journal.pone.0103257

**Published:** 2014-08-01

**Authors:** Ashwini Y. Ramdasi, Ravi P. Arya, Vidya A. Arankalle

**Affiliations:** Hepatitis Division, National Institute of Virology, Pashan, Pune, Maharashtra, India; University of North Carolina School of Medicine, United States of America

## Abstract

High mortality in pregnant women (PR) is a characteristic of hepatitis E in developing countries. To understand the pathogenesis of HEV infection in self-limiting disease during pregnancy, we compared clinical (PR-patients) and subclinical-HEV-infections in pregnant women in the first (SC-PR-1) and later (2nd and 3rd, SC-PR-2+3) trimesters with the respective healthy controls and acute non-PR patients. The SC-PR-2+3 exhibited lower ALT, bilirubin levels, anti-HEV-IgM/IgG titres than the acute-PR/non-PR-patients (p<0.05–0.0001). IFNγ/IL4ratios indicated Th2/Th1 bias in non-PR and PR-patients respectively. Raised levels of 10/20 plasma cytokines in the non-PR-patients reflect predominant inflammatory response, unaltered- IFNγ/reduced-IFNα responses and a robust chemokine secretion. On contrary, the acute-PR-patients exhibited drastic reduction in majority of the cytokines relative to in the non-PR-patients. Importantly, diminished or unaltered response was noted in the acute-PR-group when compared to the corresponding controls. The only exception was sIL2RA, increasing in both patient categories. Of the 14 genes evaluated, the expression of IFNγ/IL10/IL1A/IL7/CCL2/CCL3/CXCL8/CXCL10 was higher in the non-PR patients. Of these, the expression of IFNγ/IL10/IL1A/CCL2/CCL3/CXCL8 and, additionally, IL2/IL6/TNF genes was higher in the clinical-PRs. Almost identical pattern was noted in the control-PR-2+3 category indicating no influence of HEV infection. Comparison of patient-categories identified significant elevation of IFNγ(P<0.001), CCL2(p<0.01), CXCL8(P<0.05), IL1B(p<0.05) and IL10(P<0.0001) and decrease in CXCL10(<0.05) in the PR-patients. The results suggest antibody-dependent disease severity and impaired immune response in the PR patients. Higher expression of cytokine-genes in the PBMCs did not correlate with the plasma-cytokine levels in the PR-patients.

## Introduction

Pregnant women (PR) are at increased risk of both morbidity and mortality from a variety of viral infections such as CMV, SARS, Varicella Zoster and influenza [1]–[4]. Hepatitis E, an epidemic as well as sporadic disease prevalent in the developing countries is characterized by high mortality in pregnant women, increasing with the pregnancy trimester [5]–[8]. In the sporadic setting, men and non-pregnant women succumb to fulminant hepatitis E [9].

During pregnancy, the maternal immune system is modified to accommodate the foetus. The unique changes in hormone levels include the huge production of human chorionic gonadotropin (hCG), a placental glycoprotein, which is supposed to influence the immune system [10]. hCG induces the production of progesterone and estrogen during early pregnancy and drive the immunologic alterations both at the foeto-maternal interface and in the systemic circulation.

Pathogenesis of fulminant hepatitis E in pregnant women is poorly understood [11]–[13]. One of the important factors is the absence of severe liver disease in the pregnant rhesus monkeys, the widely used and accepted animal model for hepatitis E [14]–[15]. We have two important observations as far as hepatitis E during pregnancy is concerned. Firstly, in accordance with the reports of high mortality, in the sporadic setting, we did observe 80% (4/5) mortality during the third trimester whereas one each in the first and second trimesters survived [11]. Secondly, during a common-source epidemic of hepatitis E at Karad, in addition to the high mortality in pregnant women, we showed that a large number of pregnant women in the third trimester develop subclinical infections, the ratio of clinical: subclinical infections being 1∶13 [16].The investigation included >800 pregnant women demonstrating that despite being a high risk category, majority of these women do develop subclinical infection or self-limiting clinical disease. These observations suggest that pregnancy is not the sole important factor for the fulminant and fatal outcome of Hepatitis E virus (HEV) infection. If a large number of HEV-infected pregnant women in the third trimester clear the infection, it is important to understand the factors determining differential outcomes of HEV infection, i.e., subclinical infection, uneventful clinical disease and fulminant hepatitis leading either to recovery or death. It was thought logical to first investigate the milder forms of the disease, generate data that will form basis for a comprehensive comparison with the fulminant disease and the outcome, death v/s recovery.

This study reports preliminary analysis of the association of anti-HEV titres, cytokine profile in the plasma and mRNA levels in the PBMCs of pregnant women presenting with subclinical or clinical HEV infection with uneventful recovery.

## Materials and Methods

### Ethics Statement

The study was approved by the “Institutional Human Ethics Committee”, National Institute of Virology. The National Institute of Virology is invited by various state governments/local health authorities to investigate epidemics of viral diseases, including hepatitis. For any research component during epidemics of hepatitis E, a written informed consent is obtained from all the study subjects by the local health authorities/National Institute of Virology. The healthy pregnant women were bled on the request of the health authorities for the identification of IgM-anti-HEV positives so that they can be monitored for the symptoms and severity of the disease.

Table 1 provides details of the study population. Diagnosis of hepatitis E was based on the presence of anti-HEV-IgM antibodies in ELISA [17] and only IgM-anti-HEV positives were included in the study. The patient categories included (a) non-PR hepatitis E patients during the acute (n = 36, non-PR-patients) and (b) convalescent (n = 18, non-PR-convalescent) phases of the disease (c) pregnant women in the 2^nd^ and 3^rd^ trimester of pregnancy suffering with acute hepatitis E (PR-patients, n = 17). The mean duration of the onset of clinical symptoms and blood collection was 7.2+0.7 days (acute, non-PR), 9.7+2.3 days (acute, PR) and 36.1+3.3 days (convalescent, non-PR). The subclinical categories included, (d) subclinical HEV infections among pregnant women in the first (n = 14, SC-PR-1) and (e) 2nd and 3rd trimesters (n = 32, SC-PR-2+3). These patients were identified during 3 epidemics of hepatitis E in the rural areas of the state of Maharashtra, India (2008–2010). Two types of apparently healthy anti-HEV antibodies negative control groups included (a) Non-pregnant subjects (n = 25) and (b) Pregnant women in the first (n = 15) and later (n = 28) trimesters. Sample collection, transportation, processing and storage were identical for all the study groups. All the study subjects were screened for IgG and IgM-anti-HEV antibodies, IgM-anti-HAV antibodies, HBsAg, IgM-anti-HBc, anti-HCV and anti-HIV antibodies (ELISA, Abbott, USA). The patients were negative for the serological markers for HAV, HBV, HCV and HIV, while the controls were negative for all these markers as well as IgM and IgG anti-HEV antibodies.

**Table 1:** Characteristics of the study population*.

Categories								
Parameters	Non-PR-control (A)	PR control-1 (B)	PR control-2+3 (C)	Acute-non-PR (D)	Convalescent non-PR (E)	Subclinical-PR-1 (F)	Subclinical-PR-2+3 (G)	Acute-PR-2+3 (H)
Number	25	15	28	36	18	14	32	17
Age/Sex ratio	22±0.6/0.79∶1	22±0.7	24±0.7	29±1.8/1.57∶1	30±3.4/1∶1	23±1.0	22±0.3	23±0.7
Pregnancy (PR) status (trimester)	NA	Yes (1)	Yes (2+3)	NA	NA	Yes (1)	Yes (2+3)	Yes (2+3)
Serum ALT IU/ml (mean ± SE)	28.9±2.7	19.7±1.0	19.8±0.8	472.9±63.1	215.9±67.4	187.3±121.9	43.8±12.5	215.9±47.7
Bilirubin mg/dl (mean ± SE)	0.41±0.04	0.22±0.02	0.19±0.01	4.3±0.9	2.5±1.1	0.3±0.1	0.5±0.2	4.4±1.1
Prolactin ng/ml (mean ± SE)	ND	49.3±17.3	100.6±15.4	ND	ND	102.2±14.4	127.6±11.4	104.3±17.23
Beta HCG m IU/ml (mean ± SE)	ND	66706.4±23414.1	25880.4±5453.6	ND	ND	40530.4±11213.7	17433.1±2384.9	17729.2±2268.1
Progesterone ng/ml (mean ± SE)	ND	24.2±5.1	45.8±4.3	ND	ND	20.7±4.3	52.0±4.1	41.3±3.1
Serum Protein gm/dl (mean ± SE)	6.4±0.1	5.2±0.2	5.3±0.2	6.0±0.1	6.1±0.2	5.8±0.3	5.6±0.1	4.8±0.2
Serum Albumin gm/dl (mean ± SE)	3.4±0.1	2.3±0.1	2.1±0.1	2.8±0.1	2.9±0.1	2.7±0.2	2.3±0.1	1.9±0.1

**p Value among groups.**

**Serum ALT**: D>A (p<0.0001), F>B (p<0.05), H>C (p<0.0001)), G>C (p<0.05), D>E (p<0.05), D>H (p<0.01), H>G (p<0.0001).

**Bilirubin**: D>A (p<0.0001), E>A (p<0.0001), G>C (p<0.01), H>C (p<0.0001), D>E (p<0.05), H>G (p<0.0001).

**Prolactin**: C>B (p<0.05), F>B (p<0.05).

**Beta HCG**: B>C (p<0.05), F>G (p<0.05), F>H (p<0.05).

**Progesterone**: C>B (p<0.001), G>F (p<0.0001).

**Serum Protein**: D>H (p<0.0001).

**Serum Albumin**: A>D (p<0.0001), F>B (p<0.05), D>H (p<0.0001).

*The levels of creatinine, urea and globulins were within normal range for all the groups.

NA-Not applicable; ND-Not done.

A detailed clinical examination was done for all the AVH cases. All AVH-E patients had typical symptoms of acute viral hepatitis, such as sudden onset of fever, nausea, vomiting, weakness and jaundice. A subclinical case was defined as an IgM anti-HEV positive with or without elevated ALT levels, no typical symptoms of jaundice and no development of symptoms upto 2 months follow-up.

### Anti-HEV antibodies and biochemical parameters

The titres of anti-HEV antibodies were determined by two-fold dilutions of the sera and testing in ELISA [17]. Liver function (Bilirubin total/conjugated/unconjugated, Protein/Albumin/Globulin, AST/ALT), kidney function (Creatinine/Urea) tests and the levels of pregnancy hormones, i.e., Human Chorionic Gonadotropin (Beta-HCG), Progesterone and Prolactin were determined in the plasma samples employing Dimension RxL Max (Siemens Healthcare, USA) and Architect (Abbott, USA) respectively.

### Cytokine measurements

Plasma cytokines and chemokines (referred mainly as cytokines) levels were determined using 22-Bio-Plex Protein Array System (Bio-Rad, Herculus, CA, USA) using Milliplex Map Kit according to manufacturer's instructions For statistical analysis, a value of 0.2 pg/ml was used for samples showing undetectable concentrations.

### Immune response gene expression analysis

Gene expression analysis was done from the frozen PBMCs according to the protocol described previously [18]. Relative gene expression values were calculated using comparative Ct method using Life Technologies (USA) Relative Quantification (RQ) Manager Software v1.2 and >2.5 fold difference was considered significant. c-DNA from healthy non-pregnant controls were used as calibrators. 18SrRNA was used as endogenous control. Mean RQ values were calculated for each study group. For Cluster analysis relative quantitation values were log2 transformed and hierarchically clustered with analysis software (Cluster 3.0).

### Statistical Analysis

The Mann-Whitney U test was used for group comparisons. For cytokine analysis, significance level for p-value was adjusted to 0.007 using Bonferroni correction. For other analyses, a P value of less than 0.05 derived from a two-tailed-test was considered significant. All statistical analyses were performed with ‘SPSS11.0 for Windows’ software (SPSS Inc.). Association between ALT levels and antibody titres/cytokine levels was determined by computing Karl-Pearson correlation coefficient (r). For this, log values were used and magnitude greater than 0.5 were considered valid.

## Results

### Patient characteristics and biochemical parameters

Table 1 provides the details of the study groups. As there was no difference between the male and female controls as well as patients (data not shown), the non-PR category included both genders. Non-PR and PR-controls exhibited normal liver and kidney functions.ALT levels were higher in the non-PR-patients than the PR-patients (p<0.01) that in turn was higher than the SC-PR-2+3 category (p<0.0001). The SC-PR-1 group exhibited higher ALT levels than in the controls (p<0.05). Bilirubin levels were raised and comparable among PR and non-PR-patients and normal in both the subclinical groups. The levels of creatinine, urea and globulins were within the normal range in all the groups examined. The albumin levels were lower in non-PR-patients and SC-PR-1 group than in the corresponding controls while the levels decreased significantly in the PR-patients than in the non-PR patients.

Irrespective of the disease status, prolactin and progesterone levels were higher in the later trimesters than the first trimester (P<0.05 and 0.001 respectively) while HCG levels were lower (p<0.05) (table 1). The prolactin levels were significantly higher in subclinical HEV infection during the first trimester of pregnancy than in the corresponding controls (p<0.05).

### Anti-HEV antibody titres

**Figure 1:**
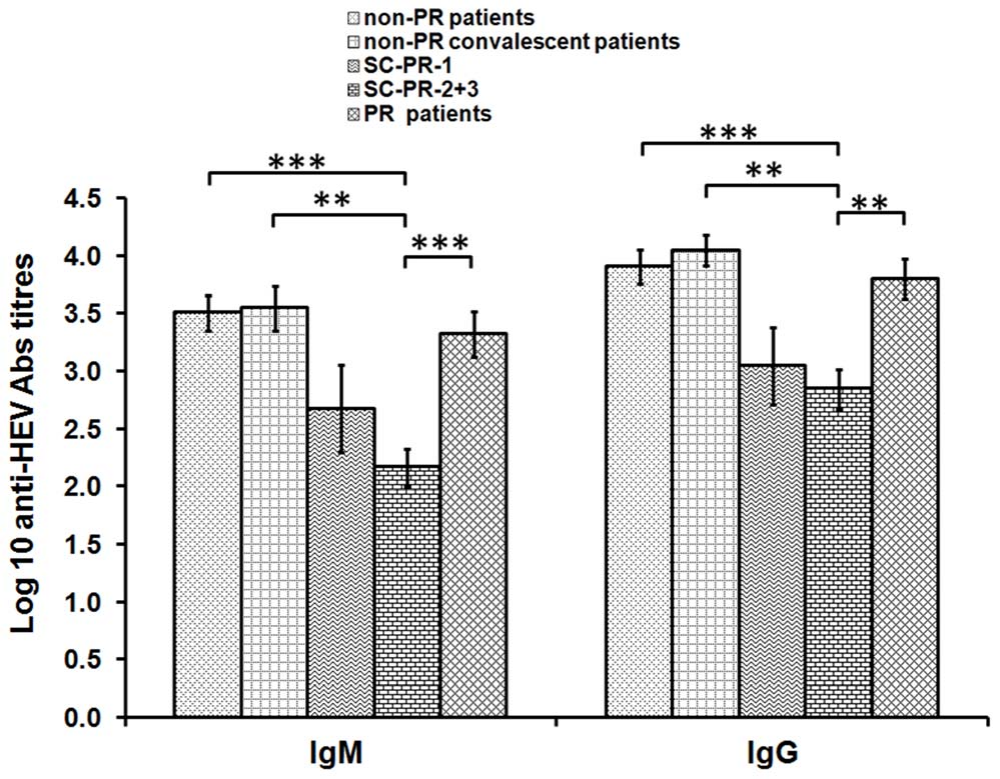
Bar diagram depicting IgM and IgG-anti-HEV titres in different categories of HEV-infected individuals. p values are indicated by stars, ** p<0.05, ***p<0.001.

Titres of IgM and IgG anti-HEV antibodies were not different among acute non-PR and PR patients (p>0.1, figure 1). When clinical and subclinical HEV infections during the later trimesters were compared, both IgM and IgG antibody titres were significantly lower in the subclinical category (p<0.005 and 0.05 respectively). Both antibody titres were significantly lower in SC-PR-2+3 than the non-PR-convalescent group (p<0.005).

### Plasma cytokines

**Figure 2:**
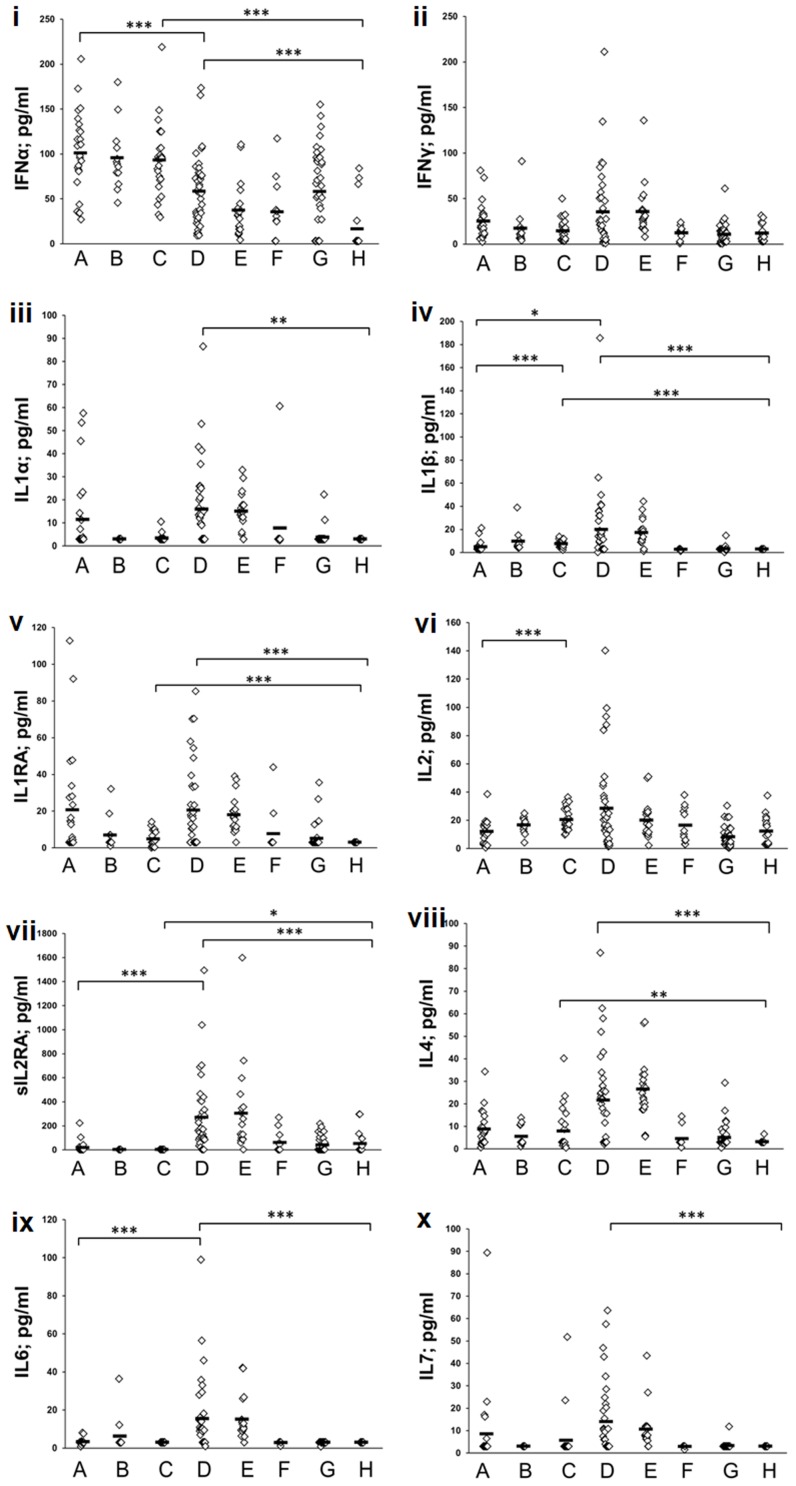
Mean cytokine levels (pg/ml) in the plasma of study subjects belonging to different categories. (A = non-pregnant controls, B = control-pregnant trimester1, C = control-pregnant trimester-2+3, D = acute non-pregnant patients, E = convalescent non-pregnant patients, F = subclinical pregnant trimester 1, G = subclinical pregnant trimester-2+3, H = acute pregnant trimester-2+3 patients). p values are indicated by stars, *p<0.007, **p<0.001, ***p<0.0001.

**Figure 3:**
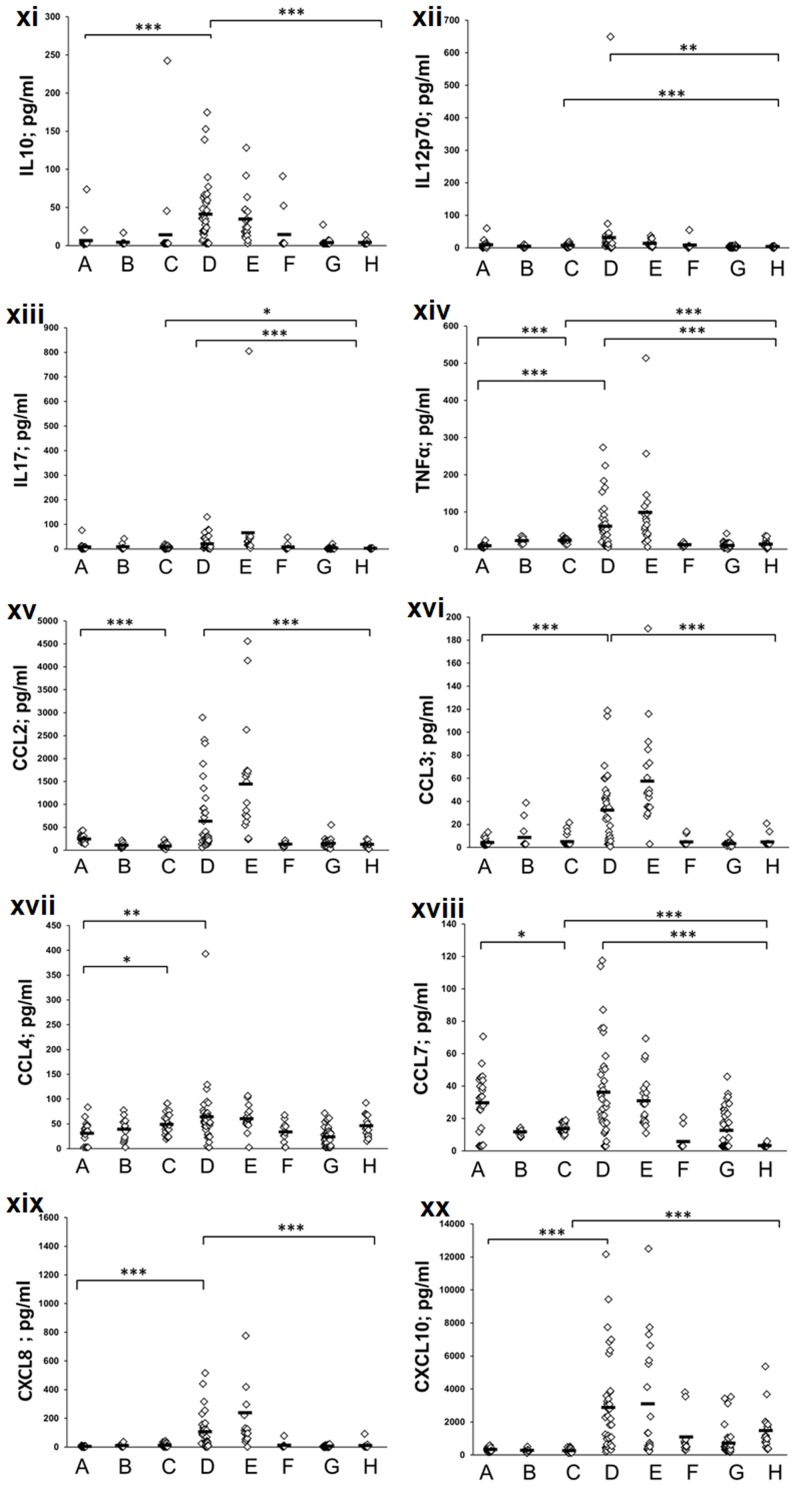
Mean cytokine levels (pg/ml) in the plasma of study subjects belonging to different categories. (A = non-pregnant controls, B = control-pregnant trimester1, C = control-pregnant trimester-2+3, D = acute non-pregnant patients, E = convalescent non-pregnant patients, F = subclinical pregnant trimester 1, G = subclinical pregnant trimester-2+3, H = acute pregnant trimester-2+3 patients). p values are indicated by stars, *p<0.007, **p<0.001, ***p<0.0001.

**Figure 4:**
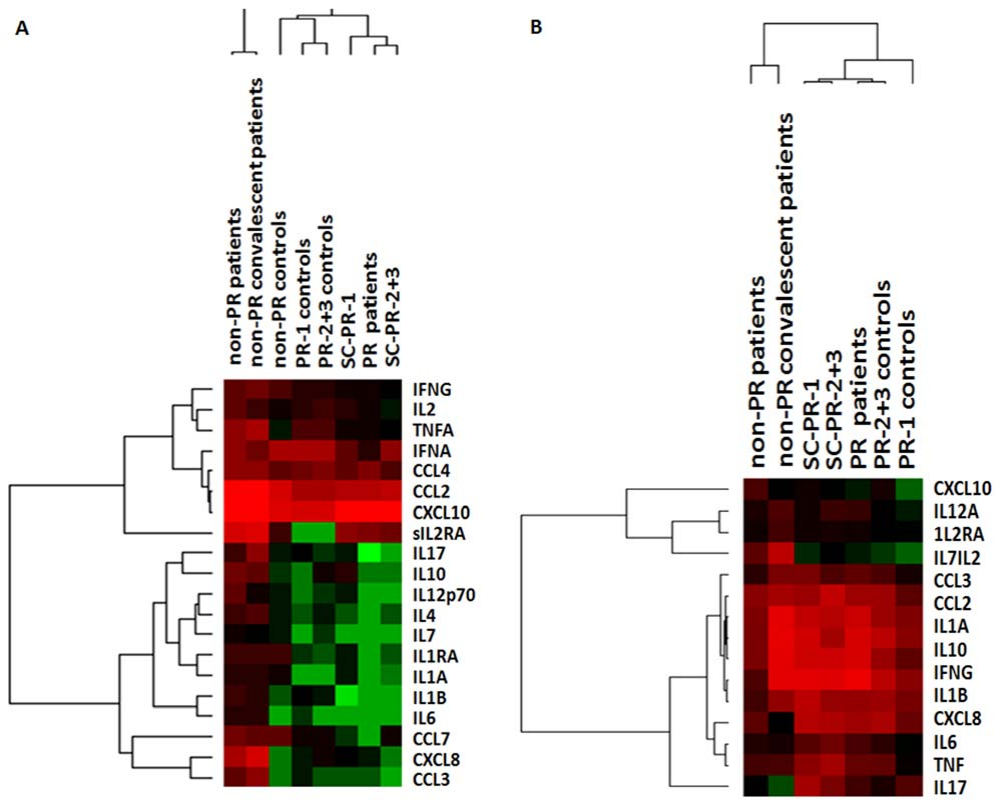
Heat maps showing cytokine patterns in different study groups at (A) protein and (B) gene expression levels. Values for 20 cytokines and 15 genes were hierarchically clustered on log2 transformation.

To identify potential differences among the non-PR and PR categories, we quantitated 20 plasma cytokines during the acute and convalescent phases in the non-PR patients and compared with clinical and subclinical HEV infections among PRs and corresponding trimester-matched controls (figures 2, 3 and 4A).

### Effect of pregnancy on the plasma cytokine levels in the control women

Throughout the healthy pregnancy, the levels of CCL2 (p<0.0005) and CCL7 (p<0.001) were significantly reduced while a substantial increase was noted for TNFα and IL1b (p<0.0005), when compared to the non-PR-controls. IL2 (<0.0001) and CCL4 (p<0.007) were significantly higher during the later trimesters. The levels of IFNγ/sIL2RA/IL1A/IL1RA/IL4/IL6/IL10/IFNα/IL12/IL17A/IL7/CCL3/CXCL8 and CXCL10 were not different in the control pregnant women during the first as well as later trimesters (p>0.007).

### Distinct patterns of cytokine production in the non-PR patients and PR categories

#### Th1/Th2 cytokines

Initial comparisons with respective controls documented no increase in IFNγ levels in both non-PR and PR-patients (p>0.1). As against no change in the non-PR group, diminished IL12 levels were noted in the PR-patients and SC-PR-2 category (p<0.0001). In the non-PR-patients, IL4 levels remained unaltered while IL10 levels increased (p<0.0001). IL10 was unaltered in all the HEV-infected pregnant women whereas lower levels of IL4 were recorded in the PR-patients (p<0.007). IL2 was unaltered in the clinical categories (p>0.01) and reduced in SC-PR-2+3 infections (<0.0001).When non-pregnant and pregnant patients were compared, a reduced secretion of IL12, IL10 and IL4 was noted in latter (p<0.0001).

Next, we compared Th1/Th2 (IFNγ/IL4) ratios among different categories (table 2). Th1 bias (ratio = 3.02) was seen in the non-PR-controls and during the first trimester-controls (ratio = 4.3). The PR-controls in the later trimesters exhibited a shift to Th2-bias (ratio = 2.1). In the non-PR-patients, both cytokines increased while the ratio decreased to 1.7 suggesting Th2 response that was enhanced during the convalescent phase (ratio = 1.3). As IL4 levels diminished in HEV-infected pregnant women, accurate determination of Th1/Th2 bias was not possible. However, a Th1 bias was evident.

**Table 2:** Th1/Th2 (IFNγ/IL4) ratios in different categories.

Categories	pg/ml		IFNγ/IL4 ratio
	IFNγ	IL4	
Non-PR-control	25.1	8.3	3.02
PR control-1	17.3	4.0	4.3
PR control-2+3	14.5	6.7	2.1
Acute-non-PR	34.9	20.8	1.7
Convalescent non-PR	35.7	26.5	1.3
Acute-PR-2+3	11.3	0.56	20.2
Subclinical-PR-2+3	10.1	3.31	3.1
Subclinical-PR-1	12.1	2.4	5.0

#### Proinflammatory cytokines and chemokines

The levels of TNFα, IL1B and IL6 were higher in the non-PR-patients (p<0.007–0.0001) while TNFα, IL1B and IL17 levels reduced in the PR-patient and PR-SC-2 groups (p<0.007–0.0001). In both patient and SC-PR-2 categories, IFNα levels were reduced (p<0.007–0.0001) while a significant reduction was noted for IL1RA in clinical and subclinical infections during later trimesters (p<0.0005). All these cytokines were reduced in PR-patients than in the non-PR patients (p<0.007–0.0001).

The levels of CCL3/CCL4/CXCL8/CXCL10 were significantly higher in the non-PR-patients than in the controls (p<0.0005) while CCL2 and CCL7 were not different (p>0.1). In the PR-patients, CCL3/CCL4 levels were unchanged (p>0.1), CXCL10 was raised (p<0.0001) whereas CCL7 (p<0.0001) was reduced. SC-PR-2+3 group had significantly lower CCL4 (p<0.001) and higher CCL2 (p<0.0001), CXCL10 (p<0.001) levels. Except CCL4 and CXCL10, all the other chemokines were reduced in PR-patients than in the non-PR-patients.

sIL2RA levels increased in all the HEV-infected individuals (p<0.007–0.0001), except PR-SC-1 group. No difference was observed in the IL1A levels. When clinical categories were compared, sIL2RA and IL1A levels were reduced in patients with pregnancy (p<0.0001).

### Correlation of ALT rise with antibody titres/cytokine levels

We further analysed correlation of ALT levels (liver damage) with antibody titres and cytokine levels. ALT rise correlated with IgM-anti-HEV titres (r = 0.61, p<0.0001) and CXCL10 (r = 0.79, p<0.0001), IL10 (r = 0.6, p<0.0001), sIL2RA (r = 0.512, p<0.0001), IL6 (r = 0.507, p<0.0001) levels.

### Gene expression pattern in the PBMCs from different categories

Of the 14 genes evaluated, the expression of IFNγ, IL10, IL1A, IL7, CCL2, CCL3, CXCL8 and CXCL10 was higher in the non-PR-patients (≥2.5 fold, table 3).Of these, the expression of IFNγ, IL10, IL1A, CCL2, CCL3, CXCL8 and additionally, IL2, IL6 and TNF genes were higher in the clinical-PRs. However, almost identical pattern (except IL6) was noted in the control-PR-2+3 category indicating no influence of HEV infection on the pregnancy-induced alterations in the gene expression pattern.

**Table 3:** Cytokine gene expression fold changes (RQ values) in different categories (mean±SE).

Genes	Non-PR patients-A	PR patients-B	PR-2+3 controls-C	SC PR-2+3-D	PR-1 controls -E	SC PR-1-F
CCL2	4.7±1.3	11.7±2.1	6.8±1.2	9.2±2.0	4.8±2.0	10.7±3.0
CXCL10	2.6±1.2	0.7±0.1	1.4±0.6	1.1±0.5	0.3±0.1	1.3±0.3
CXCL8	3.2±0.6	7.1±1.4	8.1±1.5	8.4±1.3	3.7±1.0	9.2±2.8
CCL3	5.6±2.3	6.9±2.4	6.9±1.4	11.0±2.2	3.1±0.7	6.7±1.3
IFNG	2.7±0.6	17.9±5.8	10.3±1.3	15.0±4.8	5.5±1.6	16.3±2.1
IL2	1.8±0.2	3.3±0.9	2.6±0.5	2.7±0.6	1.4±0.2	4.5±1.4
IL2RA	1.3±0.3	1.3±0.2	1.1±0.1	1.4±0.2	0.9±0.1	1.3±0.2
IL17	1.1±0.2	2.3±1.4	1.5±0.3	4.5±1.7	2.8±1.7	7.6±4.7
IL1A	4.5±1.2	13.6±6.9	10.2±1.9	7.5±1.9	5.3±1.0	12.8±4.7
IL1B	2.3±0.7	6.3±2.0	6.7±1.4	6.5±1.2	4.4±1.6	9.9±3.8
IL6	1.6±0.3	2.5±0.6	1.8±0.3	3.9±0.9	0.9±0.2	3.0±1.3
IL7	3.2±1.6	0.7±0.1	0.5±0.1	1.0±0.2	0.3±0.0	0.6±0.1
TNF	2.4±0.4	3.5±1.3	3.4±0.9	7.5±1.6	1.2±0.3	5.6±1.8
IL10	4.6±1.0	14.0±3.9	6.6±1.0	12.2±2.7	3.3±0.9	12.4±2.1

Significant p values among groups.

**CCL2**: B>A (p<0.01), B>C(p<0.05).

**CXCL10**: B>A (p<0.05).

**CXCL8**: **B**>A (p<0.05).

**IFNG**: B>A (p<0.001).

**IL1B**: B>A (p<0.05).

**TNF**: C<D (p<0.05).

**IL10**: B>A (p<0.05), D>C(p<0.05), B>C(p<0.05).

Comparison of the patient categories identified significant elevation of IFNγ (P<0.001), CCL2 (p<0.01), CXCL8 (P<0.05), IL1B (p<0.05) and IL10 (P<0.0001) and decrease in CXCL10 levels (<0.05) in the PR-patients. No difference was noted when PR patients and SC-PR-2+3 group were compared. In the control-PR-1 category, the expression levels of CCL2, CCL3, CXCL8, IFNγ, IL17, IL1A, IL1B and IL10 were increased while CXCL10 and IL7 were lower. No difference was observed when healthy pregnant (1and 2+3 trimesters) control groups were compared.

Comparison of heat maps for cytokines at protein and gene levels (figure 4) clearly differentiated non-PR and PR-categories. In the PR-patients, a distinct pattern, i.e., significant reduction at protein levels as against elevated/normal gene expression was evident. Except for the down-regulation of IL7 and CXCL10 in the PR-1 controls, the other genes in all the study groups were either up-regulated or expressed at normal levels, irrespective of the cytokine levels (table 4).

**Table:** **Table 4.** Association of plasma cytokine and PBMC gene expression levels in different categories.

Cytokine	non-PR patients		PR patients		PR 2+3 controls		SC PR 2+3		PR-1 Controls	
	Plasma	PBMC	Plasma	PBMC	Plasma	PBMC	Plasma	PBMC	plasma	PBMC
IFNG	normal	↑	↓	↑	normal	↑	↓	↑	normal	↑
IL2	normal	normal	normal	↑	↑	↑	normal	↑	normal	normal
sIL2RA	↑	normal	normal	normal	normal	normal	normal	normal	normal	normal
IL17	normal	normal	↓	normal	normal	normal	↓	↑	normal	↑
IL1A	normal	↑	↓	↑	normal	↑	↓	↑	normal	↑
IL1B	↑	normal	↓	↑	↑	↑	normal	↑	↑	↑
IL6	↑	normal	normal	↑	normal	normal	↓	↑	normal	normal
IL7	↑	↑	normal	normal	normal	normal	normal	normal	normal	↓
TNF	↑	normal	normal	↑	↑	↑	normal	↑	↑	normal
IL10	↑	↑	normal	↑	normal	↑	normal	↑	normal	↑
CCL2	normal	↑	↓	↑	↓	↑	↓	↑	↓	↑
CCL3	↑	↑	normal	↑	normal	↑	normal	↑	normal	↑
CXCL10	↑	↑	↑	normal	normal	normal	↑	normal	normal	↓
CXCL8	↑	↑	↑	↑	normal	↑	normal	↑	normal	↑

↑ = Elevated as compared to non-PR controls (p<0.007). Normal (plasma): comparable with non-PR controls; normal (PBMCs):<2.5fold change when normalized with the non-PR controls.

## Discussion

Host factors are known to play crucial role in the outcome of infections. In order to understand the pathogenesis of HEV infection during pregnancy, we investigated self-limiting, subclinical and clinical HEV infections among pregnant women. Subclinical infections in the later trimesters were characterized by lower ALT and bilirubin levels and exhibited reduced anti-HEV-IgM and IgG titres. (table 1, figure 1). The possibility of HEV infection in the subclinical cases being much earlier than the clinical cases was ruled out as the antibody titres in the subclinical cases were significantly lower than even convalescent non-PR-patients. The clinical disease in both categories led to comparable anti-HEV antibody titres. Earlier, we reported a significant increase in the titres of IgM/IgG-anti-HEV antibodies in the PR and non-PR fulminant hepatitis E patients when compared to the patients with uneventful recovery [11]. Taken together, these findings reveal a clear relationship of antibody titres with severity of hepatitis E and suggest antibody-mediated liver damage. Importantly, a significant increase in the HEV antigen-specific, IgG antibody-producing B cells in patients with FHF-E than in the uncomplicated hepatitis E patients has been shown [19].

Cytokines, the important immunologic messenger molecules, are secreted in the blood stream and have multiple direct/regulatory functions depending on the infecting pathogens [20]. Though most relevant, studying the affected organ is impractical, especially for self-limiting infections and blood remains the specimen of choice providing useful information. Variable cytokines are detected in individuals without apparent acute or chronic infection and these steady state cytokines are known to differ in urban and rural populations [21]. We have tried to minimize these variables as the both control and patient populations came from the same villages and communities.

It is believed that pregnancy is associated with a systemic shift toward a Th2 cytokine profile [22]. However, a prospective study [23] failed to demonstrate this effect. Comparison of IFNγ/IL4 ratios suggested Th2 bias during the later trimesters of healthy pregnancy and non-PR patients that increased during convalescent phase. Despite diminished levels of IL4 in pregnant patients, a Th1 bias was evident. In this context, our earlier observation of high cytokine levels secreted by the HEV-specific-antigen-stimulated PBMCs coupled with a Th2 bias in 3/4 fatal FHF-E in pregnant women in the later trimesters [11] is noteworthy. Though we have determined plasma cytokines, the results suggest Th1 to Th2 shift in fulminant disease during pregnancy. A partial attribution of shift from Th1 to Th2 to the development of acute lung injury in the pregnant rats infected with influenza virus is noteworthy [24].

Our results are partially at variance from a study in patients from north India probably because the authors have grouped all the pregnant women without mentioning trimesters [25]. Though Acute-PR patients in both studies exhibited Th1 response whereas as against higher antibody titres and shift to Th2 response in fulminant hepatitis E noted by us [11], a stronger Th1 response was observed by Borkakoti et al [26]. Similarly, as against low/absence of viral load in the FHF patients from our series, a high viral load was observed [26]. The reasons for these differences are not clear, except for different population types examined.

The study generated for the first time, data on the levels of 20 plasma cytokines in healthy pregnant women from western India. We showed that the healthy pregnancy was associated with a significant lowering of CCL2/CCL7 and increase in TNF/IL1b levels while IL2 and CCL4 increased during the later trimester. The pattern seems to vary with the population types [27]–[28].

The disease in pregnancy was distinctly different with respect to the circulating cytokine levels, 16/20 cytokines were reduced in pregnant patients than the non-PR-patient group. sIL2RA was the only cytokine raised in all HEV infected individuals (except SC-PR-1). As blood cytokines have multiple effects on different circulating cell types, it seems logical that the PR-patients may have dysregulated/impaired immune response. A further significant decrease in CXCL10, CCL4 and rise in IFNα, CCL7 levels differentiated pregnant women presenting with subclinical and clinical HEV infections respectively. Low levels of IFNγ even in the non-PR patients is noteworthy.

The systemic cytokine changes in HEV infection in the non-PR patients reflect predominant inflammatory response, unaltered/reduced antiviral cytokines such as IFNγ/IFNα respectively and a robust chemokine secretion. On contrary, the PR-patients predominantly exhibited diminished or unaltered response. The only exception was sIL2RA (sCD25) showing increase in both patient categories when compared to the controls suggestive of enhanced Treg activity [29]. Earlier, based on the elevated levels of peripheral CD4^+^CD25^+^Foxp3^+^ and CD4^+^+CD25^−^Foxp3^+^ cells in non-pregnant hepatitis E patients [30], we suggested involvement of regulatory T cells (Treg) in hepatitis E. Further, the Treg cells were shown to be functional and could suppress/inhibit autologous effector T cells [31]. Taken together, the results suggest that HEV infection during pregnancy is associated with elevated levels of Treg cells.

Though IFNα levels were independent of healthy pregnancy and decreased in HEV infected pregnant women in the later trimesters, the levels were higher in subclinical category, whether the higher levels direct asymptomatic infection needs to be explored. Correlation of ALT levels with IgM-anti-HEV titres and rise in four cytokines, CXCL10, IL10, sIL2RA and IL6 demonstrate association of these cytokines with ongoing liver damage in acute HEV infection.

When we compared different cytokines at protein and gene levels, discordance between gene expression and protein levels was evident in HEV-infected pregnant women in the later trimesters. As proposed by Keene [32], the role of an infrastructure between the genome and the proteome termed a ribonome may tightly regulate the early response genes such as cytokines.

In conclusion, the anti-HEV antibody titres were directly proportional to disease severity in pregnant women. The disease in pregnancy was associated with a significant reduction in the plasma cytokines despite increase in the corresponding gene expression in the PBMCs. The study needs to be extended to fulminant hepatitis E for the understanding of the pathogenesis of this form of infection with high mortality.
